# Autonomic Inertia as a Proximal Risk Marker for Moments of Perseverative Cognition in Everyday Life in Remitted Depression

**DOI:** 10.1155/da/9193159

**Published:** 2024-12-23

**Authors:** Sarah L. Zapetis, Jiani Li, Ellie P. Xu, Zihua Ye, Coralie S. Phanord, Timothy J. Trull, Stefan Schneider, Jonathan P. Stange

**Affiliations:** ^1^Department of Psychology, University of Southern California, Los Angeles, California, USA; ^2^Department of Psychology, University of Illinois at Urbana-Champaign, Champaign, Illinois, USA; ^3^Department of Psychology and Neuroscience, University of Colorado Boulder, Boulder, Colorado, USA; ^4^Department of Psychological Sciences, University of Missouri, Columbia, Missouri, USA; ^5^Center for Economic and Social Research, University of Southern California, Los Angeles, California, USA; ^6^Department of Psychiatry and Behavioral Sciences, University of Southern California, Los Angeles, California, USA

**Keywords:** ambulatory psychophysiology, autonomic complexity, depression, ecological momentary assessment, perseverative cognition

## Abstract

**Background:** Trait perseverative cognition (PC) is associated with inflexible autonomic activity and risk for depressive recurrence. However, the identification of dynamic psychophysiological markers of PC that fluctuate within individuals over time could facilitate the passive detection of moments when PC occurs in daily life.

**Methods:** Using intensively sampled data across 1 week (3x/day) in adults with remitted major depressive disorder (rMDD) and never-depressed controls (CONs), we investigated the utility of monitoring ambulatory autonomic complexity to predict moments of PC engagement in everyday life. Autonomic complexity metrics, including the root mean square of successive difference (RMSSD), indexing vagal control, and sample entropy, indexing signal complexity, were calculated in the 30 min *before* each measurement of PC to enable time-lagged analyses. Multilevel models examined proximal fluctuations in the mean level and inertia of complexity metrics as predictors of subsequent PC engagement.

**Results:** Momentary increases in the inertia of sample entropy, but not other metrics, predicted higher levels of subsequent PC in the rMDD group, but not among never-depressed CONs.

**Conclusions:** The inertia of sample entropy could index autonomic rigidity and serve as a dynamic risk marker for real-world PC in individuals with a history of depression. This could inform the development of technologies to passively detect fluctuations in risk for PC, facilitating real-time interventions to prevent PC and reduce the risk for depressive recurrence.

## 1. Introduction

Depression is a pervasive psychiatric disorder and a leading cause of disability worldwide [1]. The high rate of recurrence following remission from a depressive episode is an important contributor to this public health challenge [2, 3]. Accordingly, there is a pressing need for research to enhance our understanding of factors that contribute to the onset and recurrence of depression. One key risk factor is perseverative cognition (PC), characterized by the persistent and repetitive activation of negative thoughts [4, 5]. Engagement in PC, which has been conceptualized as a way of responding to negative affect [6, 7], fluctuates throughout the day, reflecting the dynamic interplay between emotions and cognitive processes. As such, just-in-time adaptive interventions (JITAIs) that can identify and promptly intervene during moments of heightened risk for PC may be particularly well-suited to target this cognitive risk factor. Several interventions, like mindfulness-based cognitive behavioral therapy (CBT) and rumination-focused CBT, have been developed to target PC and have shown some promise in reducing rates of depressive recurrence [8, 9]. However, the real-world implementation of these intervention techniques is currently limited by our inability to detect moments when PC occurs, precluding intervention deployment in moments when it could be most beneficial. Moreover, identifying moments of heightened risk for PC engagement in daily life could enable the implementation of intervention strategies during critical moments to prevent PC. Utilized in combination with existing interventions, this approach has the potential to enhance their efficacy. Thus, the goal of our current study was to determine whether continuously monitoring psychophysiology in daily life could facilitate the detection of moments of increased PC engagement.

PC can be divided into two key components: rumination, which involves engaging with negative material from the past, and worry, which involves a focus on negative events that might occur in the future [4, 6, 7, 10]. Empirical evidence consistently indicates that trait rumination predicts the onset, duration, and severity of major depressive episodes [11–15]. Moreover, research indicates that worry serves as an important risk factor for depression as well [16, 17]. Specifically, trait levels of worry have been shown to predict the recurrence and persistence of depressive disorders as well as the severity of depression symptoms [5]. Importantly, both rumination and worry are thought to prolong cognitive representations of stressors, either retrospectively or in anticipation, along with stress-related affect and physiological activation that can contribute to dysregulation and disease [4]. Furthermore, high trait PC has been linked to impairments in inhibitory control and cognitive flexibility, reflected in difficulties shifting cognitive resources to changing situational demands [18, 19], which may contribute to challenges inhibiting or disengaging from these putatively maladaptive cognitive responses to stress. Thus, despite differences in their temporal content, rumination and worry are both types of PC that are difficult to control and are highly correlated with one another [16, 20, 21].

Whereas a substantial body of literature has examined trait-like PC, typically assessed with one time, self-report measures in which participants rate their usual engagement in PC [22, 23], limited research has used intensive sampling methods to examine how PC fluctuates within individuals over time. Given the dynamic nature of emotional experiences and cognitive responses to emotions in our daily lives, as well as the susceptibility of retrospective measures to recall biases [24, 25], research designs that utilize in-the-moment data to capture within-person variability are needed to aid in the development of more ecologically valid models of affect dysregulation [26]. Ambulatory assessment technologies offer a way to intensively sample an individual's momentary state within their everyday life to investigate fluctuations in constructs of interest. Whereas measures like ecological momentary assessment (EMA) require persistent effort from participants, ambulatory psychophysiological measures can be passively and unobtrusively collected from wearable devices and therefore may be more feasible for long-term risk monitoring. To the extent that people's physiology changes in the moments before they engage in PC, ambulatory psychophysiology has the potential to allow for the identification of moments *when* individuals are at acute risk for PC in everyday life. In turn, this detection could facilitate the implementation of in-the-moment intervention strategies to prevent real-world PC engagement.

Autonomic complexity is a promising psychophysiological construct that could serve as a proximal marker for moments of risk for real-world PC. Autonomic complexity can be derived from measures of variation in the time between consecutive heartbeats (e.g., heart rate variability) and may reflect the capacity of the autonomic nervous system to respond flexibly to changing demands [27]. Neurovisceral integration theory posits that, during rest, the prefrontal cortex exerts parasympathetic inhibitory control over thalamic regions associated with sympathetic defensive responses via the vagus nerve [28–31]. This enables the autonomic nervous system to be responsive to input from a variety of internal and external stimuli, which manifests as slowed heart rate and greater variability in the intervals between heartbeats (greater autonomic complexity). Conversely, PC may correspond to a failure of these inhibitory processes resulting in prolonged physiological arousal, as evidenced by lower resting autonomic complexity in individuals with high trait PC [32, 33].

Having higher levels of autonomic complexity at rest is thought to signify the integration of neural inhibitory circuits that support adaptive regulation of affect and physiology. In contrast, reductions in autonomic complexity during stress or emotional challenges may represent a contextually appropriate withdrawal of this inhibitory control that enables activation of sympathetic defensive responses. The ability of the autonomic nervous system to flexibly modulate responses to changing situational demands—as reflected in fluctuations in autonomic complexity across contexts—is critical for effective emotion regulation [31, 34–36]. For example, in a prior investigation, autonomic inflexibility in response to a laboratory sadness induction was associated with higher levels of trait PC, as well as greater engagement in real-world PC regardless of stress exposure [37]. As such, inflexible autonomic activity may reflect vulnerability to context-insensitive engagement in PC and could serve as a marker of individual differences in risk for depression [38]. However, *at the within-person level*, little research has examined whether moments of risk for PC in daily life are characterized by short-term decreases in autonomic flexibility. Moreover, recent evidence has highlighted the importance of investigating intraindividual variability in autonomic complexity metrics over time in real-world contexts [27, 39]. As such, a key aim of this study was to investigate fluctuations not only in the *level* of complexity but also the *inertia—*a novel dynamic metric of autonomic sluggishness—as predictors of proximal fluctuations in real-world PC.

Inertia represents the temporal dependency of a signal or how strongly two consecutive measurements are associated with each other [40, 41]. Our novel application of inertia to autonomic complexity builds upon an existing literature on *emotional inertia*, which indicates that the inertia of negative emotions is elevated in depression [42, 43] and is positively associated with rumination across individuals [44], suggesting that negative emotions are more resistant to change in depression. At the physiological level, a recent ambulatory study found that across individuals, reduced variability of autonomic complexity across a week of measurement was associated with higher levels of negative affect and ruminative brooding [27]. However, to our knowledge, the *inertia* of autonomic complexity metrics as a momentary indicator of proximal fluctuations in PC *within* individuals has yet to be investigated. This investigation would more precisely address our clinical interest in detecting moments *when* PC is likely to occur. In our study, greater inertia would reflect more similarity or sluggishness in autonomic complexity metrics within the intervals just prior to the measurement of PC in everyday life (Figure S1).

A commonly used index of autonomic complexity is the root mean square of successive difference (RMSSD) between heartbeats. RMSSD is a linear time-domain measure that approximates vagally mediated influences on the heart [45]. Therefore, higher resting RMSSD reflects greater integration of parasympathetic inhibitory circuits in the regulation of heart rate. Whereas linear measures like RMSSD are often used to estimate physiological processes, the autonomic nervous system is influenced by a variety of regulatory mechanisms, and thus its output is nonlinear in nature [46]. As such, nonlinear measures like sample entropy may be better suited to capture autonomic complexity and may reflect shifts in the balance between the sympathetic and parasympathetic branches of the autonomic nervous system. Sample entropy is a computation of signal irregularity that captures the degree to which short-term patterns in a signal are predictable over time (Figure S2). While low levels of sample entropy indicate that a system is more repetitive, showing consistent patterns across time, higher levels may indicate that a system has more sources of input and is more flexible to changing demands [47].

Given trait-level associations between PC and reduced autonomic flexibility in both laboratory paradigms [37] and daily life [27], we hypothesized that periods of *decreased level* and periods of *increased inertia* of autonomic complexity metrics would predict subsequent increases in EMA-reported PC. In our sample of adults with a history of remitted major depressive disorder (rMDD) and never-depressed controls (CON) individuals, we also examined depression history as a moderator of the within-person relationships between fluctuations in autonomic complexity and PC hypothesizing that, relative to CONs, individuals with rMDD would show a stronger relationship between maladaptive autonomic complexity metrics (i.e., decreased mean and increased inertia) and subsequent engagement in PC. This hypothesis was based on research showing that individuals with depression exhibit habitual engagement in PC, characterized by a higher degree of automaticity in response to negative affect [48], as well as impairments in cognitive flexibility that may influence the ability to inhibit or disengage from maladaptive cognitive responses to physiological arousal or rigidity in everyday life.

## 2. Materials and Methods

### 2.1. Participants

Study procedures were approved by the University of Illinois at Chicago (UIC) and the University of Southern California (USC) Institutional Review Boards. All research protocols were carried out in accordance with the provisions of the World Medical Association Declaration of Helsinki. This study involved data collected from 36 young adults with rMDD and 30 never-depressed CONs. The inclusion of individuals with rMDD allowed for the investigation of relationships of interest in a population that is at an increased risk for depressive recurrence while minimizing the influence of factors associated with the state of current depression (e.g., symptoms). In addition, it allowed for the examination of how depression history influences relationships between autonomic complexity metrics and PC. Participants were 18–30 years old which served to minimize the cumulative effects of recurrent depressive episodes, which would presumably be more prevalent in older samples. Participants were 36.4% male and 63.6% female, with a mean age of 26.7 years (standard deviation [SD] = 3.8). Participants were recruited from the surrounding community using online advertisements. All participants were required to have fluency in English and normal or corrected-to-normal vision and were excluded if they had known cardiac arrhythmias. After providing informed consent, participants were screened with the Diagnostic Interview for Genetic Studies [49]. Participants in the rMDD group met Diagnostic and Statistical Manual of Mental Disorders, Fifth Edition (DSM-5) criteria for lifetime MDD [50], met criteria for full remission for at least 8 weeks, and scored a 7 or below on the 17-item Hamilton Depression Rating Scale (HDRS) [51]. CON participants did not meet current or past criteria for MDD or any other psychiatric disorder and had no first-degree family members with known psychiatric disorders. Groups did not differ on demographic characteristics, although as expected, participants in the rMDD group had higher rates of some comorbidities and psychotropic medication use (Table 1).

### 2.2. Procedures

#### 2.2.1. EMA

Following the screening visit, eligible participants were trained on the EMA procedures. Specifically, a study staff member reviewed the EMA schedule, each EMA item, and its associated response options with participants to ensure sufficient understanding. Then, over the course of 7 days, EMA survey links were sent to participants via text or email six times a day. Surveys were presented and collected in Research Electronic Data Capture (REDCap) [53]. Participants chose to receive either an “early” schedule (i.e., surveys were sent between the hours of 8:00 am and 7:00 pm) or a “late” schedule that was offset by 2 h (i.e., surveys sent between 10:00 am and 9:00 pm) depending on their schedule preference. Within the “early” and “late” groups, the EMA surveys were sent in a fixed schedule across participants. To facilitate the appearance of a semirandom schedule, participants were not told of the specific survey schedule beyond that they would receive surveys in the morning, afternoon, and evening. The six surveys were sent in pairs with three “pre” and three “post” surveys per day. For each pair, the post-survey was sent 30 min after completion of the pre-survey. If the pre-survey was not completed, the participant was not sent a post-survey for that time point. Participants were given up to 1 h to complete each survey and were reminded every 20 min until each survey was completed, resulting in a 30- to 90-min period between pre- and post-surveys. The mean between-person duration of time between pre- and post-surveys was 46.5 min (SD = 7.1 min), indicating that on average people completed the post-survey within 15 min of receiving the initial prompt.

In all surveys, participants answered prompts about their affect based on how they felt just before the survey. In each post-survey, participants also reported their engagement in ruminative brooding and worry “since the pre-survey about 30–90 min ago” on a scale from 1 (not at all) to 10 (very much) using items adapted for EMA from the Spontaneous Affect Regulation Scale (SARS) [27, 54–56]. Specifically, ruminative brooding was measured with the item “I thought about a recent situation, wishing it had gone better” and worry was measured with the item “I worried about something.” Responses to ruminative brooding and worry items were averaged at each time point to provide a composite PC score. This PC score, while novel, demonstrated moderate reliability in our sample (*ω*_between_ = 0.701, *ω*_within_ = 0.613 [57]).

#### 2.2.2. Ambulatory Psychophysiology

During the 7-day EMA period, participants wore a lightweight biometric Hexoskin smart shirt (Carré Technologies, Inc.) under their usual clothing during waking hours to allow for the continuous collection of electrocardiogram (ECG) data in their daily lives. The shirts contain a single-lead ECG with three electrode sensors sampled at 256 Hz. The shirts also continuously collected three-axis accelerometer data sampled at 64 Hz which was used to control for activity level in models using autonomic complexity metrics as predictors.

Sequential interbeat intervals were extracted from the Hexoskin shirts and entered into HRVanalysis software [58]. Signals from each recording were visually inspected and quality checked, such that any gaps of visibly noisy portions at the beginning or end of the recording were removed before processing the signal. For segments of missing or exceptionally messy signals that were noticeable, the R–R series was split into sections and analyzed individually. The software detected and corrected anomalies due to heartbeat rhythm disturbances such as ectopic beats.

**Figure 1:**
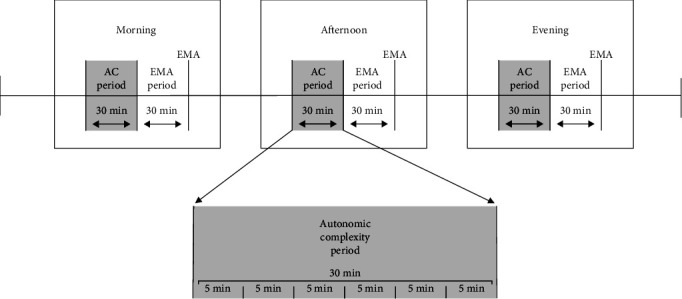
Daily ambulatory assessment procedure. *Note:* EMA post-surveys captured engagement in PC “since the pre-survey about 30–90 min ago.” ECG data in the 30 min before each EMA period was utilized to enable a time-lagged design. The RMSSD and sample entropy of each 5-min epoch were calculated. Then, the mean level and inertia across the six consecutive 5-min epochs in each autonomic complexity period were calculated. AC, autonomic complexity; ECG, electrocardiogram; EMA, ecological momentary assessment; RMSSD, root mean square of successive differences.

The data were then analyzed in 5-min epochs [59], which are thought to reflect short-term cardiac dynamics [45] and may be better suited to detect periods of proximal risk for PC compared to longer term measurements. Epochs were excluded if they contained excessive noise or missing data, as detected by the following criteria: (1) the percentage of artifacts in the R–R time series exceeded 10%; (2) more than 5 s of data were missing from the beginning or end of the 5-min segment; or (3) more than one-third of data were missing from the middle of the 5-min segment [27]. The first autonomic complexity metric, RMSSD, was obtained by first calculating the successive time difference between each pair of heartbeats, squaring each value, averaging them, and then squaring the result [45]. The second metric calculated was sample entropy, which is defined as the logarithmic likelihood that two sequences of R–R intervals that are similar for *m* time points differ at time *m* + 1 [60, 61]. Two sequences are determined to be matched if their distance is within a certain threshold *r*. In our study, *m* was set to 2 and *r* to 0.2 times the SD of the time series, which are standards commonly reported in the literature [62]. Time stamps were used to match the autonomic complexity epoch values with the corresponding EMA survey data. For the analysis of psychophysiological data, data in the 30 min *before* each EMA period were utilized to enable time-lagged analysis (Figure 1). For each 30-min autonomic complexity period, the mean and inertia of RMSSD and sample entropy were calculated using the six 5-min epochs within that period. Inertia was defined as the Pearson autocorrelation between the six consecutive epochs within a given autonomic complexity period and their lagged counterparts (Figure S1).

Physical activity levels served as a covariate in analyses that included autonomic complexity metrics as a predictor. This was done to account for metabolically driven changes in autonomic complexity [63], since psychologically driven changes were of interest in this study. Activity levels were calculated as the vector of acceleration (square root of the sum of squares of each of the three accelerometer axes) per second and averaged across each 5-min epoch. Then, the mean level of activity across each 30-min autonomic complexity period was calculated.

### 2.3. Statistical Analysis

All analyses were conducted in Mplus Version 7 [64] and utilized full information maximum likelihood (FIML) estimation to account for missing data. Sensitivity analyses were conducted to examine the effects of relevant covariates including age, gender, and body mass index (BMI) on the relationships of interest. In within-person analyses, including those with group as a moderator, additional sensitivity analyses were conducted to examine the effect of including average activity level and the average of the corresponding autonomic complexity metric across the study period.

#### 2.3.1. Group Comparisons

Groups (rMDD and CON) were compared to determine differences in repeated-measures ambulatory variables (autonomic complexity metrics and PC). Multilevel models included group as a between-person (Level 2) predictor of random intercepts (average levels) of autonomic complexity metrics and PC across observation windows.

#### 2.3.2. Within-Person Analyses

Multilevel models were estimated to examine relationships between within-person fluctuations in autonomic complexity metrics (in the 30 min *prior* to the EMA period) and the degree of engagement in PC *during* the subsequent 30-min EMA period. Autonomic complexity metrics and activity level were centered around each person's mean. Separate models were conducted for each of the four autonomic complexity metrics (mean of RMSSD, inertia of RMSSD, mean of sample entropy, inertia of sample entropy). If models including the inertia of autonomic complexity metrics indicated that inertia significantly predicted PC, sensitivity analyses were then conducted that included both the inertia and mean level of the respective autonomic complexity metric to determine the incremental effect of inertia above and beyond the effect of mean level.

To investigate how depression history impacted the strength of the within-person relationships between autonomic complexity metrics and subsequent PC, group (rMDD or CON) was examined as a between-person (Level 2) moderator of the within-person relationships between fluctuations in autonomic complexity metrics and subsequent PC, while also including the main effect of group as a predictor of PC. Significant interactions between group and autonomic complexity metrics were probed by testing simple slopes in each group [65].

#### 2.3.3. Between-Person Analyses

To determine whether participants with lower (and more inert) autonomic complexity would report more PC across the week of measurement, each autonomic complexity metric and physical activity level were averaged across the week of observation for each participant (~21 time points, linked to the 30-min windows before EMA periods), providing a single value for each person. These autonomic complexity metrics and activity level were then z-scored across participants to aid in interpretation and entered as Level 2 predictors of intercepts of PC. To investigate how depression history impacted the strength of the between-person relationships between autonomic complexity metrics and PC, group (rMDD or CON) was examined as a moderator.

#### 2.3.4. Comparisons of Inertia Metrics With Different Time Lags

Our primary analysis plan was to calculate the inertia of autonomic complexity metrics in successive (nonoverlapping) 5-min epochs, as is standard when calculating emotional inertia using EMA data [41]. However, we also conducted sensitivity analyses to determine how using overlapping epochs, with varying degrees of overlap (e.g., 50% overlap with prior 5-min epoch), would alter the autocorrelations and the predictive ability of the inertia variables. This analytical approach is described in the Supporting Information, and the results of these analyses are shown in Table S1.

## 3. Results

### 3.1. Descriptive Statistics about Ambulatory Data

A total of 1268 pre/post-survey pairs were completed (*M* = 19.21 per participant, SD = 3.06, min = 10, max = 28). On average, participants completed 87.95% of possible “pre” survey prompts (SD = 14.39%, min = 25%, max = 100%). Among the instances where pre-surveys were completed, 91.75% of possible “post” surveys (SD = 9.47%, min = 47.62%, max = 100%) were completed. Of the post-surveys completed, 26.97% were missing the corresponding psychophysiological data (i.e., the mean of autonomic complexity metrics could not be calculated) due to no data being recorded (e.g., the shirt was not worn, or device was not charged). For an additional 5.21% of completed post-surveys, the inertia of autonomic complexity metrics could not be calculated due to an insufficient amount of data (i.e., less than three consecutive epochs) within the autonomic complexity period. As a result, a total of 32.18% of completed post-surveys were missing the inertia of autonomic complexity metrics. Intraclass correlation coefficients (ICCs; Table 2) indicate the presence of both within- and between-person variability in EMA and mean level autonomic complexity variables. However, the ICCs of the inertia of autonomic complexity variables were notably low, indicating that a majority of the variance in these variables exists within individuals and highlighting the importance of measuring these variables repeatedly over time.

### 3.2. Group Comparisons

Individuals with rMDD had higher PC than never-depressed CONs (*B* = 0.706, standard error [SE] = 0.296, 95% confidence interval [CI] = [0.127, 1.286], *p*=0.017). However, the groups did not significantly differ on any of the autonomic complexity metrics (Table 2).

### 3.3. Within-Person Associations Between Autonomic Complexity and PC

Moments when people had higher *entropy inertia* (inertia of sample entropy) than usual were associated with higher levels of PC in the next 30 min (Table 3). Conversely, fluctuations in (1) the level of sample entropy, (2) the level of RMSSD, and (3) the inertia of RMSSD did not predict subsequent levels of PC (Table 3). These results did not differ when depression history and relevant covariates (age, gender identity, BMI, average activity level, and average corresponding autonomic complexity metric) were included in the models. In addition, although we did not control for PC at the previous time point in our main models [67], the results were also consistent when we did include this variable as a covariate (results available upon request). When both the mean and inertia of sample entropy were included as independent variables in the same model, the inertia, but not the mean, of sample entropy significantly predicted subsequent PC, and this effect remained significant when covariates were included.

**Figure 2:**
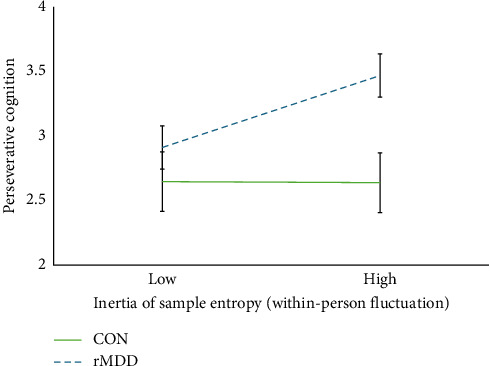
Depression history as a moderator of the relationship between within-person fluctuations in the inertia of sample entropy and perseverative cognition. *Note:* Group membership (rMDD or CON) moderated the relationship between the inertia of sample entropy and perseverative cognition at the within-person level such that individuals with rMDD exhibited a significantly positive association and CON individuals exhibited a nonsignificant association. CON, never-depressed control group; rMDD, remitted major depressive disorder group.

In the moderation analyses, we found that the within-person relationship between entropy inertia and PC differed significantly based on depression history (Table 3), with entropy inertia predicting subsequent PC in individuals with remitted depression (*B* = 0.576, SE = 0.167, 95% CI = [0.249, 0.902], *p*=0.001, *ΔR^2^ within* of the random slope of entropy inertia = 0.060, and *ΔR^2^ between* of the random slope of entropy inertia = 0.029), but not among never-depressed CONs (*B* = −0.008, SE = 0.231, 95% CI = [−0.460, 0.443], *p*=0.971; Figure 2) (this moderation was attenuated when age [*B* = 0.558, SE = 0.285, 95% CI = [0, 1.117], *p*=0.050], gender [*B* = 0.565, SE = 0.294, 95% CI = [−0.011, 1.141], *p*=0.055], and BMI [*B* = 0.557, SE = 0.286, 95% CI = [−0.003, 1.118], *p*=0.051] were included as covariates in the model. However, the simple slopes of the rMDD group were still significantly positive [age: *B* = 0.560, SE = 0.160, 95% CI = [0.246, 0.874], *p*  < 0.001; gender identity: *B* = 0.571, SE = 0.170, 95% CI = [0.238, 0.903], *p*=0.001; BMI: *B* = 0.574, SE = 0.164, 95% CI = [0.252, 0.895], p  < 0.001], whereas slopes of the CON group were nonsignificant [age: *B* = −0.003, SE = 0.228, 95% CI = [−0.451, 0.445], *p*=0.990; gender identity: *B* = 0.001, SE = 0.234, 95% CI = [−0.457, 0.459], *p*=0.997; BMI: *B* = −0.003, SE = 0.228, 95% CI = [−0.449, 0.444], *p*=0.991] when these covariates were included). The relationships between PC and (1) the mean of RMSSD, (2) the inertia of RMSSD, and (3) the mean of sample entropy did not differ based on depression history.

### 3.4. Between-Person Associations Between Autonomic Complexity and PC

**Figure 3:**
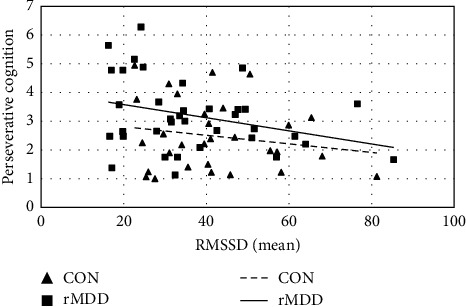
Between-person association between the mean of RMSSD and perseverative cognition. *Note:* The mean of RMSSD was negatively associated with perseverative cognition in the full sample such that individuals with a lower mean RMSSD engaged in more perseverative cognition. Group did not significantly moderate this relationship, indicating that the slope of this association did not significantly differ between groups. CON, never-depressed control group; rMDD, remitted major depressive disorder group; RMSSD, root mean square of successive differences.

**Figure 4:**
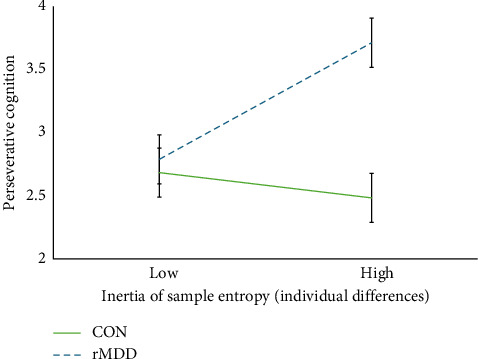
Depression history as a moderator of the relationship between the inertia of sample entropy and perseverative cognition at the between-person level. *Note:* The slope of the between-person relationship between the inertia of sample entropy and perseverative cognition differed significantly by group with rMDD individuals exhibiting a significantly positive association and CON individuals exhibiting a nonsignificant association. CON, never-depressed control group; rMDD, remitted major depressive disorder group.

At the between-person level, the mean of RMSSD was negatively associated with PC (*ΔR*^2^ = 0.081, Table 4 and Figure 3) such that individuals with a lower mean RMSSD engaged in more PC overall. This association remained significant when covariates were included in the model. Conversely, (1) the inertia of RMSSD, (2) the mean, and (3) inertia of sample entropy were not dimensionally associated with PC at the between-person level. However, the relationship between entropy inertia and PC differed significantly by group (Table 4 and Figure 4), indicating that *people* with more inertia tended to perseverate more, but only if they had remitted MDD (rMDD group: *B* = 0.484, SE = 0.204, 95% CI = [0.085, 0.884], *p*=0.017; CON group: *B* = −0.105, SE = 0.203, 95% CI = [−0.503, 0.292], *p*=0.603) (this moderation was no longer significant when age was included as a covariate in the model [*B* = 0.426, SE = 0.287, 95% CI = [−0.137, 0.988], *p*=0.138]. However, the simple slope of the rMDD group was still significantly positive [*B* = 0.518, SE = 0.191, 95% CI = [0.144, 0.892], *p*=0.007], and the simple slope of the CON group was nonsignificant [B = 0.092, SE = 0.211, 95% CI = [−0.321, 0.505], *p*=0.662] when this covariate was included). The associations between (1) the mean and (2) inertia of RMSSD and (3) the mean of sample entropy did not significantly differ based on depression history.

## 4. Discussion

This study explored links between ambulatory autonomic activity and subsequent engagement in PC during daily life in individuals with and without a history of depression. Notably, this study is the first to examine nonlinear autonomic complexity metrics in daily life as they relate to proximal fluctuations in EMA-reported PC. By investigating temporally lagged relationships within individuals—rather than between individuals, as has been typically examined in the past—we aimed to gain a better understanding of psychophysiological factors temporally linked with PC. The identification of a proximal marker for real-world engagement in PC that can be passively assessed with wearable devices could facilitate risk monitoring and inform the development of preventative tools. We found that periods characterized by higher entropy inertia were more likely to be followed by greater engagement in PC in individuals with a history of depression. We found similar relationships at the between-person level, such that individuals with remitted depression who exhibited higher inertia of sample entropy across the measurement period engaged in more PC on average. This was not the case for never-depressed CON individuals. Taken together, these results suggest that entropy inertia may serve as a novel ambulatory metric with utility for identifying both *who* may be at an increased risk for engagement in PC, and perhaps more importantly, moments in daily life *when* risk for engagement in PC is elevated, in individuals with a history of depression.

The observed within-person coupling between higher entropy inertia and increased engagement in PC partially supports our initial hypotheses. While we predicted that this coupling would be present to some degree in both groups and would be stronger in individuals with a history of depression, we only observed a significant association in the clinical group. Given the lack of group differences in entropy inertia, this finding suggests that individuals with a history of depression may be more likely than never-depressed individuals to respond to increases in entropy inertia by engaging in PC. However, it is also possible that the reduced range of PC engagement in the CON group, compared to the rMDD group, may have obscured certain associations that could be evident had a broader range of PC been sampled in both groups.

The entropy inertia metric in this study is a measure of sluggishness in autonomic signal complexity and may represent real-world autonomic inflexibility. Whereas higher levels of signal complexity in the absence of stressors are thought to reflect the integration of sympathetic and parasympathetic influences and the potential for the system to respond flexibly [47], changes in signal complexity across time in everyday life may represent adaptive responses to shifting environmental or internal demands [68, 69]. Conversely, sluggishness in signal complexity may represent inflexibility in the balance between branches of the autonomic nervous system and reduced capacity for affect regulation [29]. This autonomic inflexibility in everyday life may align with existing literature on emotion context insensitivity in depression [70, 71]. Specifically, individuals with depression tend to exhibit a general disengagement from the environment, characterized by attenuated emotional reactivity to both positive and negative external stimuli and blunted physiological reactivity in laboratory studies [72]. Our findings may extend these results to daily life contexts, as increases in entropy inertia may represent periods of heightened disengagement from external stimuli and a shift of attentional resources to internal processes such as spontaneous thought [73]. Consequently, the increased automaticity of PC engagement observed in individuals with a history of depression [48] may increase the likelihood that spontaneous thoughts transition to PC in this population. Thus, while an increase in entropy inertia may indicate a universal shift to internally focused attention, this hypothesis offers insight into why such increases may result in increased PC engagement only among individuals with a history of depression. Future research should investigate these hypotheses to clarify whether increases in entropy inertia may represent periods of disengagement from external stimuli in everyday life and how this may contribute to heightened risk for PC in certain individuals.

Fluctuations in the other autonomic complexity metrics assessed at the within-person level, including the proximal mean and inertia of RMSSD and the mean of sample entropy, were not associated with fluctuations in PC. Given the novelty of our analyses, we did not make specific predictions about differences between autonomic complexity metrics in their relationship to PC. However, variations in the timescale of these metrics may help to account for these disparate findings. While RMSSD provides information about short-term variation by comparing only successive heartbeat intervals, sample entropy measures the likelihood that pairs of successive heartbeat intervals will exhibit similarity when an additional interval is included across the sampling window [61]. Compared to RMSSD, which is thought to mainly represent parasympathetic activity, sample entropy takes into account sequences in the heart rate signal that are both closer together and farther apart than RMSSD and is thus thought to reflect more complex information about the balance of parasympathetic and sympathetic influences. Moreover, while limited studies have explored fluctuations in linear versus nonlinear autonomic complexity in daily life, there is some evidence from trait level, laboratory findings that measures of signal complexity may provide clinically relevant information, beyond linear measures. Specifically, lower resting signal complexity, but not RMSSD, has been associated with increased subclinical symptoms of depression in a sample of young adult females [74]. In addition, a recent study found that individuals with a history of depression who exhibited lower levels of resting signal complexity while tapering off of antidepressants had a higher chance of recurrence [75]. Further, lower resting signal complexity, but not linear measures, has been associated with worse cognitive inhibition performance [46], suggesting that nonlinear metrics may be particularly sensitive to fluctuations in cognitive processes like PC. In sum, our results broadly align with existing research while identifying a novel marker of proximal shifts in PC in everyday life. This work suggests that nonlinear autonomic metrics, particularly signal complexity, may hold utility for predicting clinically relevant outcomes such as risk for depressive recurrence, which should be a focus of future research.

At the between-person level, individuals with a history of depression who had higher levels of entropy inertia exhibited a higher level of PC engagement, paralleling our findings about fluctuations at the within-person level. In some ways, this association between *trait* entropy inertia and PC may help to clarify discrepancies in lab-based findings examining autonomic reactivity in individuals with remitted depression. Specifically, some studies have found blunted autonomic reactivity to stress or emotional challenges in individuals with remitted depression compared to never-depressed CONs [76, 77], while others have found no difference [78–80]. However, limited research has investigated the role of trait-level PC in autonomic reactivity among individuals with rMDD. Our results suggest that, among individuals with a history of depression, only those who have higher levels of trait PC may exhibit entropy inertia, which may result in diluted group-level effects when combining all depressed individuals in a single group regardless of PC tendencies. While a direct parallel cannot be drawn between autonomic reactivity in laboratory paradigms and autonomic sluggishness in everyday life, future research could investigate whether individuals with rMDD that have higher trait PC are more likely to exhibit blunted autonomic reactivity than those with lower trait PC to potentially clarify the noted discrepancy. Given the heterogeneity that exists in depression [81, 82], the identification of factors—such as entropy inertia—that could confer risk of recurrence in certain subgroups of individuals with remitted depression will be important to improving prevention and treatment.

We also found that individuals with a lower level of RMSSD exhibited greater PC engagement across the measurement period. This association was not moderated by depression history, suggesting a dimensional relationship. This dimensional finding extends trait-level observations examined in laboratory research [32, 33, 83, 84] in which individuals with lower RMSSD at rest tend to show greater trait PC. Establishing parallels between laboratory and ambulatory studies may serve a dual purpose. First, it bolsters confidence in the ecological validity of laboratory findings by suggesting their real-world relevance. Further, it indicates that, in cases where ambulatory measurements are impractical, laboratory-based assessments may still provide valuable insights into trait levels of autonomic complexity as they relate to real-world PC engagement. Second, these parallels provide evidence that ambulatory tools can effectively capture relevant information about relationships that have been well-characterized in laboratory research. Overall, our finding that the trait-level association between RMSSD and PC extends to measurement in everyday life supports the robust nature of this relationship.

The results of this study may have several important clinical implications. First, the within-person temporal association between fluctuations in entropy inertia and PC highlights the potential for ambulatory psychophysiology to inform JITAI tools that can detect periods of increased risk for engagement in PC in daily life. Specifically, using real-time data and algorithms to compute proximal increases in inertia, JITAIs could provide individuals with a history of depression support during moments when their physiology suggests they may need it most, to help them prevent or inhibit PC and reduce risk for depressive recurrence. One approach to intervention could involve directly targeting autonomic processes with techniques like mobile biofeedback or biocueing [85], which aims to increase awareness and self-regulation of physiological functions. While biofeedback has shown some promise in treating depression symptoms [86], further research is needed to determine the potential of biofeedback to modify entropy inertia, and whether doing so could reduce episodes of PC. Another approach to a JITAI could involve utilizing ambulatory psychophysiology to trigger behavioral interventions known to be effective in preventing and reducing PC, such as cognitive restructuring and mindfulness strategies [87], during moments when inertia fluctuates above an individual's own usual threshold. Second, the trait-level association between entropy inertia and PC in individuals with a history of depression suggests the possibility of passively identifying individuals who tend to engage in higher levels of PC-based solely on their entropy inertia. As wearable devices become more affordable and ubiquitous [88], this passive identification approach could be integrated into broader screening initiatives to identify individuals at higher risk for depressive recurrence and connect them with resources to aid in prevention.

This study has several strengths, including the use of a novel, dynamic metric to index autonomic complexity in daily life. In addition, by investigating temporally lagged associations between fluctuations in autonomic activity and PC, this study may provide insight into factors that are temporally, and potentially even causally, linked to PC, offering valuable information for preventative measures. Another significant strength is the use of passive measurement methods, which may allow for unobtrusive monitoring of risk factors over extended periods of time. In addition to the strengths, there are several important limitations that should be considered. First, since contextual information during the measurement of autonomic complexity was not accounted for in the analyses, we cannot definitively conclude that increases in entropy inertia represent a maladaptive lack of flexibility to changing environmental demands. However, given that the autonomic complexity metrics were measured across 30-min time intervals, it seems probable that some degree of fluctuation, even in response to one's own thoughts and emotions, would be expected and adaptive during this period. Second, the implications of this study may be limited by the effect size of the primary within-person finding, as fluctuations in entropy inertia explained 6% of the variance in subsequent fluctuations in PC in the rMDD group. While this effect is modest, it is important to consider the real-world nature of this study and thus the introduction of many contextual factors that could contribute to momentary fluctuations in PC. Further, the identification of an association across modalities (i.e., between psychophysiology and EMA) minimizes the impact of shared method variance and thus may contribute to a smaller overall effect. Moreover, given that the goal of the current study was to identify proximal markers of PC, to the extent that this association can be reliably replicated, entropy inertia may have some utility for passively detecting periods of increased risk for PC engagement, despite the small effect size. To enhance the predictive capacity for PC, future research should incorporate additional passively collected measures that may further increase the variance explained. Another limitation of this study involves the restricted number of items used to measure PC. While this measure demonstrated moderate reliability in our sample, subsequent investigations should consider including additional items to capture other dimensions of PC (i.e., controllability, repetitiveness) and determine how they may be uniquely related to entropy inertia. Additionally, while we used nonoverlapping 5-min epochs to compute autonomic complexity metrics, which is standard in the field [45, 59], future research should explore how different epoch overlaps (as we examined in supplementary analyses) and epochs lengths (e.g., 1 min) impact these metrics and their associations with real-world PC. Moreover, examining these associations in active MDD could help clarify state- and trait-level factors influencing the relationship between entropy inertia and PC, potentially offering a broader range of both variables and revealing stronger or more nuanced effects with additional implications for the treatment of depression. Furthermore, considering evidence that PC engagement is elevated across many mental health conditions [89, 90], further research should investigate the specificity of the relationship between fluctuations in entropy inertia and PC to depression versus a broader risk for psychopathology transdiagnostically. Lastly, to provide additional support for establishing whether the entropy inertia–PC relationship is potentially causal in nature, subsequent investigations should examine whether and how entropy inertia can be manipulated and whether altering this metric results in changes in PC engagement.

## 5. Conclusions

In conclusion, ambulatory monitoring of entropy inertia could have utility for detecting moments of increased risk for engagement in PC. Given a wealth of evidence implicating PC engagement as a key risk factor in depression, the ability to prevent or reduce engagement in PC in individuals with a history of depression is essential to reducing the high rate of recurrence. The identification of a passive physiological risk marker of PC may allow for unobtrusive, long-term monitoring, and in-the-moment interventions that could complement conventional treatments and reduce the prevalence of depressive recurrence. Overall, this study offers insights toward understanding the complex relationship between autonomic activity and PC in daily life, offering promising avenues for future research on physiological mechanisms of PC and for intervention development.

## Figures and Tables

**Table 1:** Group differences in demographic and clinical variables.

Measure	CON	rMDD
Demographics		
Age–mean (SD)	26.2 (3.8)	27.1 (3.8)
BMI–mean (SD)	24.0 (3.3)	25.5 (5.4)
Gender identity		
Male	36.7%	36.1%
Female	63.3%	63.9%
Nonbinary	0.0%	0.0%
Race		
American Indian or Alaska native	0.0%	0.0%
Asian	26.7%	8.3%
White	50.0%	63.9%
Black or African American	20.0%	22.2%
Native Hawaiian or other Pacific Islander	0.0%	2.8%
Other	0.0%	2.8%
Hispanic or Latino	16.7%	8.3%
Educational attainment–mean (SD)	16.6 (2.3)	16.2 (1.9)
Clinical features		
Lifetime comorbid diagnoses		
Dysthymia⁣^*∗*^	0.0%	19.4%
Panic disorder	0.0%	11.1%
Agoraphobia	0.0%	2.8%
Specific phobia	0.0%	5.6%
Social phobia⁣^*∗*^	0.0%	25.0%
Generalized anxiety disorder	0.0%	2.8%
Obsessive–compulsive disorder	0.0%	2.8%
Post-traumatic stress disorder	0.0%	11.1%
Other specified/unspecified anxiety disorder	0.0%	2.8%
Binge eating disorder	0.0%	8.3%
Substance use disorder (alcohol)	0.0%	11.1%
Substance use disorder (cannabis)	0.0%	2.8%
Attention-deficit hyperactivity disorder	0.0%	5.6%
Current psychotropic medications		
Antidepressant⁣^*∗*^	0.0%	27.8%
Mood stabilizer	0.0%	2.8%
Antipsychotic	0.0%	2.8%
Anxiolytic	0.0%	5.6%
Benzodiazepine	0.0%	2.8%
Stimulant	3.3%	0.0%
Opioid	3.3%	0.0%
Total medication load⁣^*∗*^–mean (SD)	0.08 (0.37)	0.42 (0.74)

*Note:* Educational attainment is reported in years where 12 years represent a high school diploma, 16 years represent a bachelor's degree, 18 years represent a master's degree, and 20 years represent a doctoral degree. Psychotropic medication load was calculated for each person following a coding system [52], which assigns a score for each medication based on its dosage and frequency and sums scores for a total medication load.

Abbreviations: %, percent of CON or rMDD group; BMI, body mass index; CON, never-depressed control group; rMDD, remitted major depressive disorder group; SD, standard deviation.

⁣^*∗*^Indicates significance (*p*  < 0.05).

**Table 2:** Group differences in ecological momentary assessment and ambulatory psychophysiology variables.

Measure	CON	rMDD	Effect size	ICC
	Mean (SD)	Mean (SD)	Cohen's *d*	
Ecological momentary assessment				
Perseverative cognition⁣^*∗*^	2.48 (1.20)	3.19 (1.25)	−0.58	0.314
Ambulatory psychophysiology				
Mean				
RMSSD	41.99 (14.69)	36.95 (17.16)	0.31	0.520
Sample entropy	1.19 (0.15)	1.14 (0.15)	0.39	0.179
Heart rate⁣^*∗*^	79.42 (9.40)	84.75 (9.59)	0.56	0.431
Activity	0.04 (0.02)	0.04 (0.02)	0.09	0.148
Inertia				
RMSSD	0.03 (0.16)	0.03 (0.18)	0.03	0.014
Sample entropy	−0.11 (0.15)	−0.09 (0.15)	−0.10	0.025

*Note:* ICC indicates the proportion of variance in within-person variables that occurred at the between-person level.

Abbreviations: CON, never-depressed control group; ICC, intraclass correlation coefficient; rMDD, remitted major depressive disorder group; RMSSD, root mean square of successive differences.

⁣^*∗*^Indicates significance (*p*  < 0.05).

**Table 3:** Momentary autonomic complexity predicting subsequent perseverative cognition (within-person).

Predicting perseverative cognition													
Dimensional models							Moderation by group models						
Model focal predictor	*B*	SE	95% CI	*p*	*R* ^2^ within	*R* ^2^ between	Model focal predictor	*B*	SE	95% CI	*p*	*R* ^2^ within	*R* ^2^ between
**RMSSD (mean)**					0.004	0.001	**RMSSD (mean)**					0.010	0.094
Perseverative cognition (intercept)	2.868	0.154	2.567, 3.169	<0.001	—	—	Perseverative cognition (intercept)	2.479	0.200	2.087, 2.870	<0.001	—	—
Fixed effects							Fixed effects						
Within-subject (Level 1)							Within-subject (Level 1)						
RMSSD (mean)	−0.006	0.004	−0.015, 0.002	0.143	0.003	<0.001	Activity (mean)	−2.402	1.824	−5.976, 1.173	0.188	0.002	0.002
Activity (mean)	−2.490	1.815	−6.047, 1.068	0.170	0.004	0.001	RMSSD (mean)	−0.010	0.006	−0.021, 0.002	0.111	0.003	<0.001
Random effects							Between-subject (Level 2)						
Perseverative cognition (random intercept)	1.397	0.241	0.923, 1.870	<0.001	—	—	rMDD	0.713	0.284	0.157, 1.270	0.012	0.002	0.086
							RMSSD (mean) × rMDD	0.006	0.008	−0.010, 0.002	0.462	<0.001	<0.001
Random effects						
Perseverative cognition (random intercept)	1.267	0.213	0.849, 1.685	<0.001	—	—
RMSSD (mean) (random slope)	<0.001	0.006	−0.013, 0.013	0.987	0.009	0.005

**RMSSD (inertia)**					0.001	0.002	**RMSSD (inertia)**					0.004	0.094
Perseverative cognition (intercept)	2.868	0.154	2.566, 3.169	<0.001	—	—	Perseverative cognition (intercept)	2.465	0.214	1.958, 2.972	<0.001	—	—
Fixed effects							Fixed effects						
Within-subject (Level 1)							Within-subject (Level 1)						
RMSSD (inertia)	−0.009	0.103	−0.210, 0.193	0.932	<0.001	<0.001	Activity (mean)	−1.560	1.625	−4.745, 1.626	0.337	<0.001	0.006
Activity (mean)	−1.568	1.600	−4.704, 1.569	0.327	0.001	0.001	RMSSD (inertia)	0.147	0.166	−0.179, 0.473	0.376	<0.001	<0.001
Random effects							Between-subject (Level 2)						
Perseverative cognition (random intercept)	1.396	0.241	0.923, 1.869	<0.001	—	—	rMDD	0.729	0.328	0.087, 1.371	0.026	<0.001	0.084
							RMSSD (inertia) × rMDD	−0.259	0.210	−0.670, 0.153	0.218	0.002	<0.001
Random effects						
Perseverative cognition (random intercept)	1.267	0.214	0.847, 1.686	<0.001	—	—
RMSSD (inertia) (random slope)	0.017	0.004	0.009, 0.024	<0.001	<0.001	0.005

**Sample entropy (mean)**					0.005	0.001	**Sample entropy (mean)**					0.016	0.096
Perseverative cognition (intercept)	2.868	0.154	2.567, 3.170	<0.001	—	—	Perseverative cognition (intercept)	2.511	0.207	2.105, 2.916	<0.001	—	—
Fixed effects							Fixed effects						
Within-subject (Level 1)							Within-subject (Level 1)						
Sample entropy (mean)	−0.374	0.317	−0.997, 0.248	0.238	0.004	<0.001	Activity (mean)	−2.797	2.173	−7.056, 1.462	0.198	0.004	0.003
Activity (mean)	−2.929	2.216	−7.273, 1.415	0.186	0.005	0.001	Sample entropy (mean)	−0.556	0.417	−1.373, 0.262	0.183	0.004	<0.001
Random effects							Between-subject (Level 2)						
Perseverative cognition (random intercept)	1.397	0.242	0.923, 1.871	<0.001	—	—	rMDD	0.679	0.288	0.114, 1.244	0.019	<0.001	0.082
							Sample entropy (mean) × rMDD	0.389	0.436	−0.465, 1.243	0.372	<0.001	<0.001
Random effects						
Perseverative cognition (random intercept)	1.264	0.212	0.849, 1.679	<0.001	—	—
Sample entropy (mean) (random slope)	0.420	0.432	−0.428, 1.267	0.332	0.014	0.007

**Sample entropy (inertia)**					0.012	<0.001	**Sample entropy (inertia)**					0.075	0.097
Perseverative cognition (intercept)	2.867	0.154	2.566, 3.169	<0.001	—	—	Perseverative cognition (intercept)	2.643	0.210	2.231, 3.055	<0.001	—	—
Fixed effects							Fixed effects						
Within-subject (Level 1)							Within-subject (Level 1)						
** Sample entropy (inertia)**	**0.366**	**0.146**	**0.080, 0.653**	**0.012**	0.010	<0.001	Activity (mean)	−1.757	1.549	−4.793, 1.279	0.257	0.015	0.007
Activity (mean)	−1.561	1.583	−4.665, 1.542	0.324	0.001	0.001	Sample entropy (inertia)	−0.008	0.231	−0.460, 0.443	0.971	0.011	<0.001
Random effects							Between-subject (Level 2)						
Perseverative cognition (random intercept)	1.399	0.242	0.925, 1.873	<0.001	—	—	rMDD	0.547	0.282	−0.006, 1.100	0.053	<0.001	0.067
							** Sample entropy (inertia) × rMDD**	**0.584**	**0.286**	**0.025, 1.144**	**0.041**	0.002	0.001
Random effects						
Perseverative cognition (random intercept)	1.263	0.205	0.862, 1.664	<0.001	—	—
Sample entropy (inertia) (random slope)	0.554	0.166	0.228, 0.879	0.001	0.072	0.006

*Note: R^2^* for the full models represents the pseudo *R*^2^, or the proportion of variance explained by the model relative to an unrestricted (intercept-only) model containing no predictors, separately at the within- and between-person levels [66]. *R*^2^ for each predictor represents the *∆R*^2^ of given predictor when it is excluded from the full model. When interactions amongst variables were included in the full model (i.e., sample entropy [inertia] × rMDD), the computation of *∆R*^2^ for each individual predictor (i.e., sample entropy [inertia] and rMDD) involved comparing a model excluding both the interaction and the individual predictor to a model with the individual predictor added. Bolded lines indicate significant main effects in dimensional models and significant interactions in models including group as a moderator (*p* < 0.05). In these models, rMDD represents a group membership variable, where never-depressed controls were coded as 0 and individuals with remitted major depressive disorder were coded as 1.

Abbreviation: RMSSD, root mean square of successive differences.

**Table 4:** Person-level autonomic complexity predicting perseverative cognition (between-person).

Predicting perseverative cognition											
Dimensional models						Moderation by group models					
Model focal predictor	*B*	SE	95% CI	*p*	*R* ^2^ between	Model focal predictor	*B*	SE	95% CI	*p*	*R* ^2^ between
**RMSSD (mean)**					0.103	**RMSSD (mean)**					0.172
Perseverative cognition (intercept)	2.853	0.144	2.582, 3.111	<0.001	—	Perseverative cognition (intercept)	2.504	0.222	2.068, 2.940	<0.001	—
Fixed effects						Fixed effects					
Between-subject (Level 2)						Between-subject (Level 2)					
**RMSSD (mean)**	**−0.351**	**0.150**	**−0.039, −0.003**	**0.019**	**0.081**	RMSSD (mean)	−0.208	0.242	−0.683, 0.267	0.390	0.056
Activity (mean)	0.106	0.142	−7.620, 17.107	0.452	0.009	Activity (mean)	0.135	0.143	−0.145, 0.414	0.346	0.013
Random effects						rMDD	0.626	0.285	0.066, 1.185	0.028	0.066
Perseverative cognition (random intercept)	1.255	0.208	0.847, 1.663	<0.001	—	RMSSD (mean) × rMDD	−0.141	0.295	−0.719, 0.437	0.633	0.003
						Random effects					
Perseverative cognition (random intercept)	1.159	0.189	0.788, 1.529	<0.001	—

**RMSSD (inertia)**					0.023	**RMSSD (inertia)**					0.115
Perseverative cognition (intercept)	2.868	0.153	2.568, 3.167	<0.001	—	Perseverative cognition (intercept)	2.474	0.213	2.057, 2.892	<0.001	—
Fixed effects						Fixed effects					
Between-subject (Level 2)						Between-subject (Level 2)					
RMSSD (inertia)	0.032	0.175	−0.311, 0.374	0.856	0.001	RMSSD (inertia)	−0.020	0.267	−0.544, 0.503	0.939	<0.001
Activity (mean)	0.173	0.135	−0.092, 0.437	0.200	0.023	Activity (mean)	0.192	0.138	−0.079, 0.463	0.164	0.026
Random effects						rMDD	0.724	0.293	0.150, 1.297	0.013	0.091
Perseverative cognition (random intercept)	1.367	0.235	0.906, 1.829	<0.001	—	RMSSD (inertia) × rMDD	0.092	0.353	−0.599, 0.784	0.794	0.001
						Random effects					
Perseverative cognition (random intercept)	1.238	0.207	0.832, 1.643	<0.001	—

**Sample entropy (mean)**					0.024	**Sample entropy (mean)**					0.119
Perseverative cognition (intercept)	2.866	0.152	2.567, 3.164	<0.001	—	Perseverative cognition (intercept)	2.445	0.218	2.018, 2.872	<0.001	—
Fixed effects						Fixed effects					
Between-subject (Level 2)						Between-subject (Level 2)					
Sample entropy (mean)	−0.046	0.149	−0.337, 0.246	0.759	0.001	Sample entropy (mean)	0.149	0.200	−0.244, 0.542	0.458	0.001
Activity (mean)	0.155	0.153	−0.144, 0.454	0.310	0.015	Activity (mean)	0.227	0.173	−0.112, 0.566	0.190	0.029
Random effects						rMDD	0.749	0.305	0.150, 1.348	0.014	0.091
Perseverative cognition (random intercept)	1.366	0.235	0.905, 1.827	<0.001	—	Sample entropy (mean) × rMDD	−0.161	0.250	−0.651, 0.329	0.519	0.004
						Random effects					
Perseverative cognition (random intercept)	1.233	0.209	0.824, 1.641	<0.001	—

**Sample entropy (inertia)**					0.059	**Sample entropy (inertia)**					0.204
Perseverative cognition (intercept)	2.868	0.150	2.574, 3.162	<0.001	—	Perseverative cognition (intercept)	2.471	0.215	2.049, 2.892	<0.001	—
Fixed effects						Fixed effects					
Between-subject (Level 2)						Between-subject (Level 2)					
Sample entropy (inertia)	0.231	0.163	−0.090, 0.551	0.158	0.036	Sample entropy (inertia)	−0.105	0.203	−0.503, 0.292	0.603	0.030
Activity (mean)	0.148	0.134	−0.114, 0.410	0.268	0.016	Activity (mean)	0.141	0.119	−0.093, 0.375	0.237	0.014
Random effects						rMDD	0.701	0.282	0.148, 1.254	0.013	0.085
Perseverative cognition (random intercept)	1.317	0.231	0.864, 1.770	<0.001	—	**Sample entropy (inertia) × rMDD**	**0.590**	**0.282**	**0.037, 1.143**	**0.037**	**0.060**
						Random effects					
Perseverative cognition (random intercept)	1.114	0.208	0.705, 1.522	<0.001	—

*Note: R* ^2^ for the full models represents the pseudo *R*^2^, or the proportion of variance explained at the between-person level by the model relative to an unrestricted (intercept-only) model containing no predictors [66]. *R*^2^ for each predictor represents the *∆R*^2^ of given predictor when it is excluded from the full model. When interactions among variables were included in the full model (i.e., sample entropy [inertia] × rMDD), the computation of *∆R*^2^ for each individual predictor (i.e., sample entropy [inertia] and rMDD) involved comparing a model excluding both the interaction and the individual predictor to a model with the individual predictor added. Bolded lines indicate significant main effects in dimensional models and significant interactions in models including group as a moderator (*p* < 0.05). In these models, rMDD represents a group membership variable, where never-depressed controls were coded as 0 and individuals with remitted major depressive disorder were coded as 1.

Abbreviation: RMSSD, root mean square of successive differences.

## Data Availability

The data that support the findings of this study are openly available in Open Science Framework at https://osf.io/vq932/?view_only=0de9ebbd3dc64c6aa17b4859d841ea63.

## Supporting Information

Supporting Information The Supporting Information section includes descriptions and formulas for the computation of inertia and sample entropy. Additionally, it details the analytical approach of a set of sensitivity analyses investigating the impact of increasing epoch overlap on the inertia variables and the predictive ability of inertia variables with varying degrees of overlap. Figure S1. Presents a graphical representation of the inertia computation for autonomic complexity metrics. Figure S2. Presents a graphical representation of the sample entropy computation from heart rate (R–R) interval data. Table S1A. Presents the average values of inertia variables with varying degrees of overlap. Table S1B. Presents the average within-person correlation between inertia variables with varying degrees of overlap. Table S1C. Presents the primary within-person dimensional analyses replicated using inertia variables with varying degrees of overlap.
