# Supplementary material for: Autonomic Inertia as a Proximal Risk Marker for Moments of Perseverative Cognition in Everyday Life in Remitted Depression
Source: Depress Anxiety. 2024 Dec 23;2024:9193159. doi: 10.1155/da/9193159 (PMC11919009; doi:10.1155/da/9193159)
Supplement: Supporting Information — The Supporting Information section includes descriptions and formulas for the computation of inertia and sample entropy. Additionally, it details the analytical approach of a set of sensitivity analyses investigating the impact of increasing epoch overlap on the inertia variables and the predictive ability of inertia variables with varying degrees of overlap. Figure S1. Presents a graphical representation of the inertia computation for autonomic complexity metrics. Figure S2. Presents a graphical representation of the sample entropy computation from heart rate (R–R) interval data. Table S1A. Presents the average values of inertia variables with varying degrees of overlap. Table S1B. Presents the average within-person correlation between inertia variables with varying degrees of overlap. Table S1C. Presents the primary within-person dimensional analyses replicated using inertia variables with varying degrees of overlap. [file 9193159.f1.docx]

**Autonomic Inertia as a Proximal Risk Marker for Moments of**

**Perseverative Cognition in Everyday Life in Remitted Depression**

**Supplementary Materials**

**Supplementary Methods**

**Inertia Computation**

The inertia of autonomic complexity variables in this study was calculated as the lag 1 autocorrelation across the six epochs within each autonomic complexity period (See Supplementary Figure S1). See the formula below, adapted from Taylor (1990).

$$\rho\left( k \right)= \frac{\sum_{t-1}^{N-k} \left( x_{t}-\bar{x} \right)(x_{t+k}-\bar{x})}{\sum_{t-1}^{N} {(x_{t}-\bar{x})}^{2}}$$

= observation at time t

$k$ = degree of lag (k =1 in this study)

$t+ k$= serial dependency of one observation point with a specified lag

$N$ = number of observations in the time series where k < N

$x$ = mean of the total time series

**Sample Entropy Computation**

Sample entropy (SampEn) measures the likelihood that two sequences of R-R intervals that are similar over *m* timepoints differ at the next time point (*m*+1; Lanata et al., 2015; Richman & Moorman, 2000; See Supplementary Figure S2). For example, when *m* = 2, the first pair of R-R intervals is compared against all other subsequent pairs, then the second pair is compared all subsequent pairs, and so on until every duplet of R-R intervals has been compared with the rest. Then, the same procedure would be repeated for sequences of three R-R intervals (triplets). Two sequences are considered a match if the distance between them is within a threshold *r*. The proportions of matched sequences of length *m* and *m*+1 are calculated across all comparisons, and sample entropy is derived from the negative logarithm of the ratio between these two proportions. Thus, sample entropy requires three parameters to be defined: *m*, which is the embedding dimension (i.e., length) of patterns; *r*, which is the tolerance threshold for distance below which two patterns are considered matched; and *N*, which is the sample size. In our study, *m* was set to 2 and *r* to 0.2 times the standard deviation of the time series, which are standards commonly reported in the literature (Fiskum et al., 2018).

*For a 5-min epoch of R-R intervals $u\left( n \right)=u\left( 1 \right),u\left( 2 \right),\ldots,u\left( N \right)$, SampEn is calculated as follows (Richman & Moorman, 2000):*

1. *Form N-m-1 vectors (“templates”) of length m. Each pair of templates is defined as:*

$$x_{m}\left( i \right)=\left[ u\left( i \right),u\left( i+1 \right),\ldots,u\left( i+m-1 \right) \right],1\leq i\leq N-m$$

$$x_{m}\left( j \right)=\left[ u\left( j \right),u\left( j+1 \right),\ldots,u\left( j+m-1 \right) \right],1\leq ij\leq N-m, j\neq i$$

2. *Let $d\left( x_{m}\left( i \right),x_{m}\left( j \right) \right)$ be the Euclidean distance between $x_{m}\left( i \right)$ and $x_{m}\left( j \right)$.*
3. *Let $B_{i}$ denote the number of $x_{m}\left( j \right)$’s that are within r unit distance from $x_{m}\left( i \right)$, such that $d\left( x_{m}\left( i \right),x_{m}\left( j \right) \right)\leq r$.*
4. *Define $B_{i}^{m}\left( r \right)=\frac{B_{i}}{N-m-1}$, which is the probability of finding a match for the template $x_{m}\left( i \right)$.*
5. *Define $B^{m}\left( r \right)=\frac{\sum_{i=1}^{N-m} B_{i}^{m}\left( r \right)}{N-m}$, which is the average probability of finding a match for templates of length m.*
6. *Repeat the above steps with templates of length m+1. Define $A_{i}$ as the number of $x_{m+1}\left( j \right)$’s that are within r unit distance from $x_{m+1}\left( i \right)$, such that $d\left( x_{m+1}\left( i \right),x_{m+1}\left( j \right) \right)\leq r$.*
7. *Define $A_{i}^{m+1}\left( r \right)=\frac{A_{i}}{N-m-1}$, which is the probability of finding a match for the template $x_{m+1}\left( i \right)$.*
8. *Define $A^{m+1}\left( r \right)=\frac{\sum_{i=1}^{N-m} A_{i}^{m+1}\left( r \right)}{N-m}$, which is the average probability of finding a match for templates of length m+1.*
9. $SampEn\left( m,r,N \right)=-ln\left( \frac{A^{m+1}\left( r \right)}{B^{m}\left( r \right)} \right)$*, where $\frac{A^{m+1}\left( r \right)}{B^{m}\left( r \right)}$ is the conditional probability that two sequences match for m+1 data points given that they are matched for m data points.*

*If there are many length-m matches but few length-(m+1) matches, this means that most length-m sequences do not continue to match at the next timepoint. In this case, similarity of patterns does not help predict the next measurements, which suggests high irregularity of the time series. This will result in a small value of $\frac{A^{m+1}\left( r \right)}{B^{m}\left( r \right)}$ ,and consequently a large value of $-ln\left( \frac{A^{m+1}\left( r \right)}{B^{m}\left( r \right)} \right)$ (i.e., SampEn).*

**Inertia Time-Lag Analyses**

Sensitivity analyses explored the impact of increasing epoch overlap on autocorrelations and the predictive ability of inertia variables. In contrast with EMA data, continuous sampling of psychophysiological data allows for data to be segmented in windows that are either adjacent or overlapping. Upon initial examination of our data, we noticed that the grand average of sample entropy inertia was negative and significantly different from zero. This would suggest that, on average, within an individual, having higher sample entropy during one 5-minute epoch was associated with a lower sample entropy during the next 5-minute epoch. In addition, we found that the average RMSSD value was slightly positive but did not significantly differ from zero. Given that the inertia of autonomic complexity metrics, to our knowledge, has not been previously investigated, we decided to conduct additional analyses to investigate how lagging the inertia of the autonomic complexity metrics in different ways may impact their average value and their relationship with the outcome of interest (PC). Specifically, as a sensitivity analysis, we computed the inertia of autonomic complexity metrics and activity as the autocorrelation between 5-minute epochs within each 30-minute period that were 50% overlapping (where each 2.5 minutes within each 5-minute epoch overlapped with the previous 5-minute epoch) and 90% overlapping (where 4.5 of each 5-minute epoch overlapped with the previous epoch). We then investigated whether the average of these values across people differed significantly from zero and in which direction (positive or negative). Next, we calculated the average within-person correlation between inertia variables with different lags to determine whether they were providing distinct information. Lastly, we repeated the primary dimensional analyses using the overlapping inertia metrics as predictors of PC.

We found that inertia of autonomic complexity variables with larger lags were associated with larger positive average values that were significantly different from zero (**Supplementary Table S1A**). In addition, the non-overlapping inertia variables used in primary analyses were moderately correlated with inertia variables that used a 50% overlap (RMSSD: r = .670; Sample Entropy: r = .525) and were less correlated with the inertia variables with a 90% overlap (RMSSD: r = .436; Sample Entropy: r = .288; **Supplementary Table S1B**). None of the inertia variables with overlapping epochs significantly predicted subsequent PC (**Supplementary Table S1C**). Future research should continue to investigate the timescale on which fluctuations in the inertia of autonomic complexity metrics is optimal for predicting PC.

**Supplementary Figures**

**
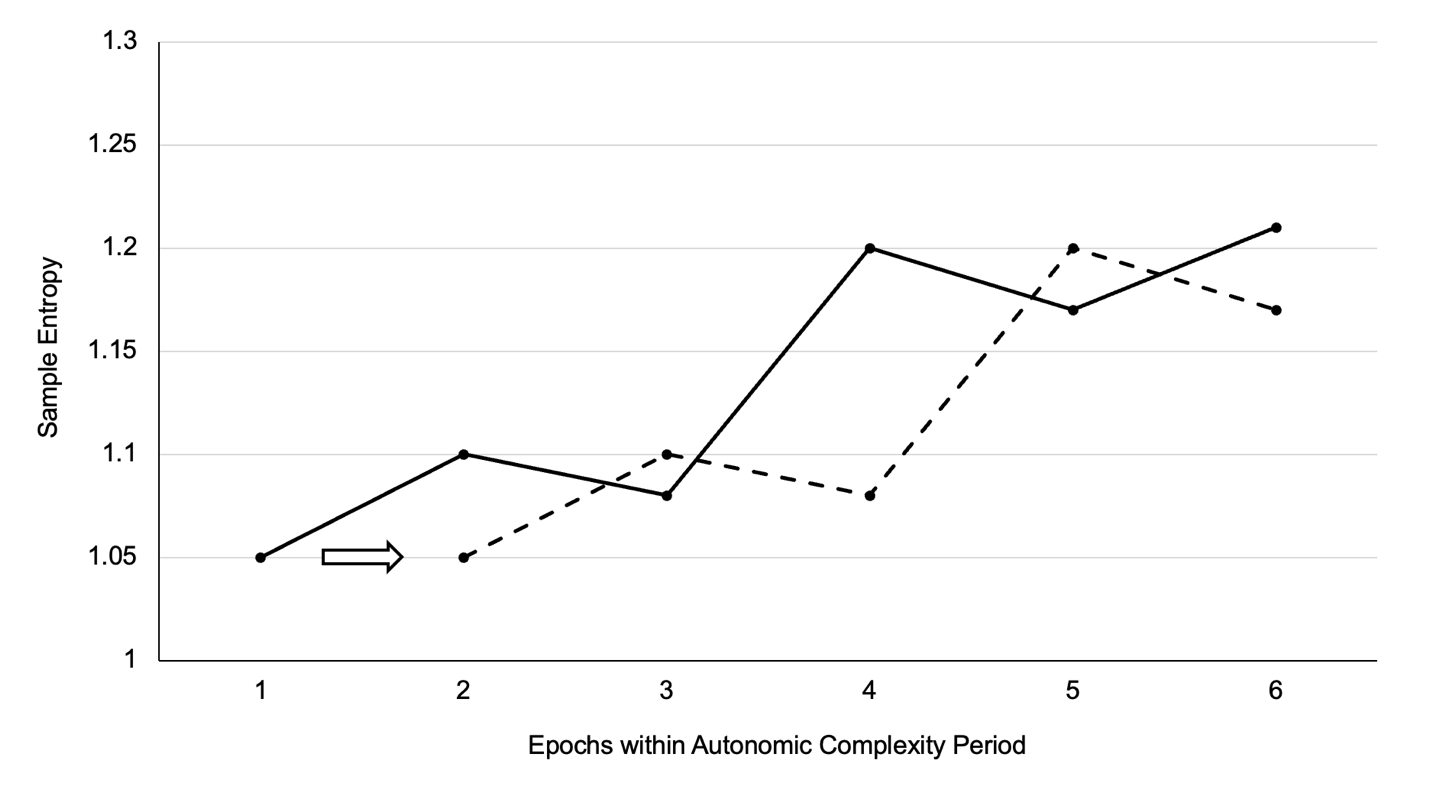
**

**Supplementary Figure S1**. **Inertia of Autonomic Complexity Metrics.** To calculate the inertia of autonomic complexity metrics, the values of RMSSD and Sample Entropy (shown here) for the six epochs within each autonomic complexity period (points connected by solid line) were lagged by 1 (points connected by dotted line). Then, the Pearson correlation between the unlagged and lagged data was computed for each autonomic complexity period.

**
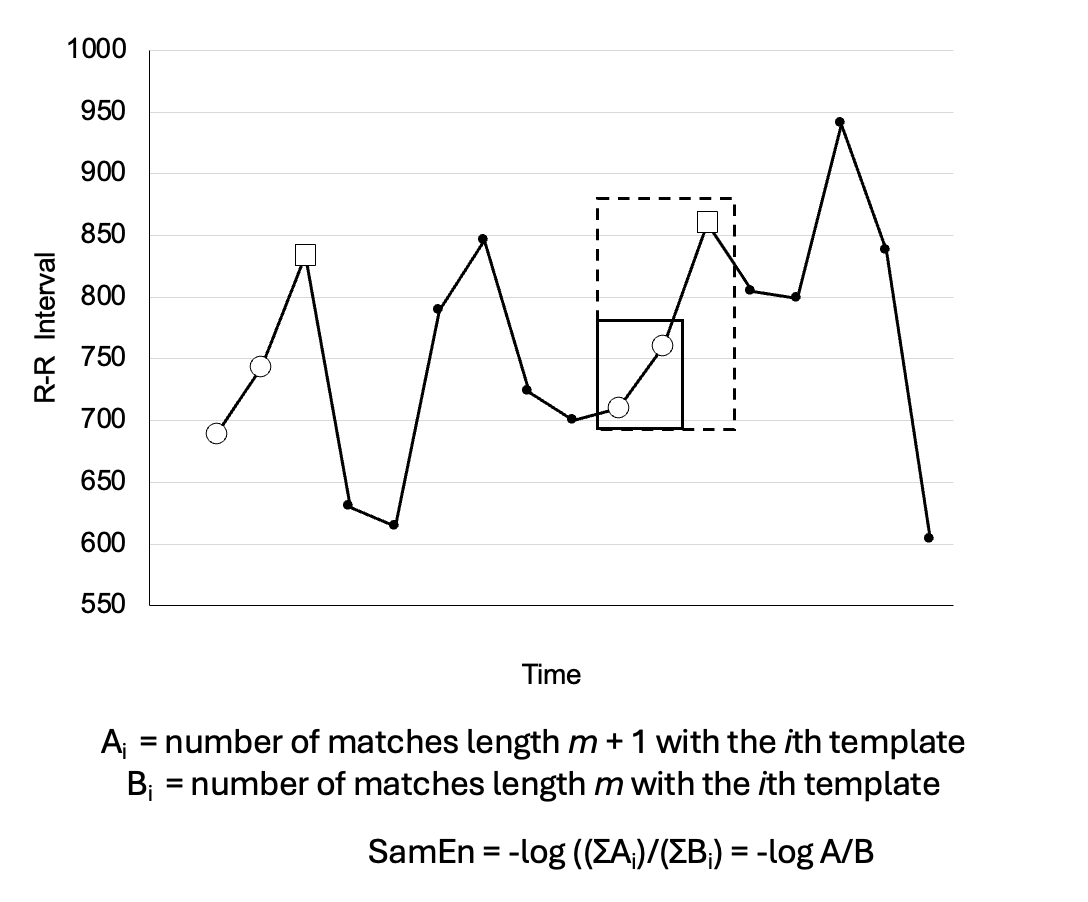
**

**Supplementary Figure S2. Sample Entropy Calculation from Heart Rate Interval Data.** The two empty circles on the left represent a template of length *m* (where *m* = 2) to which all other consecutive pairs of points in the signal are compared. The three empty shapes on the right represent a template of length *m* + 1 to which all other *m* + 1 sets of points are compared. The threshold for matching (*r*) was set to 0.2 times the standard deviation of the signal. The number of matches for each template length are determined (A_i_ and B_i_ ) and then entered into the computation for sample entropy shown at the bottom. Figure adapted from Richman et al. (2004).

**Supplementary Tables**

**Supplementary Table S1. Inertia Time-Lag Analyses**


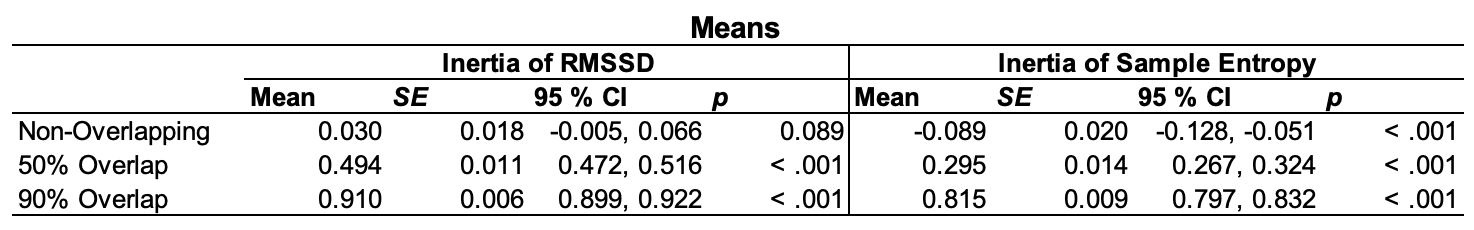
**A)**


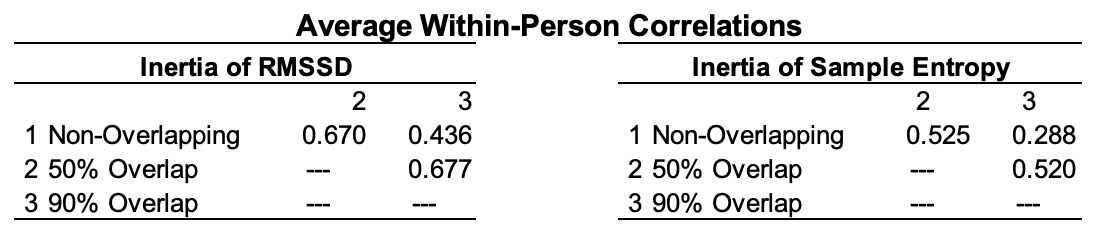
**B)**

**
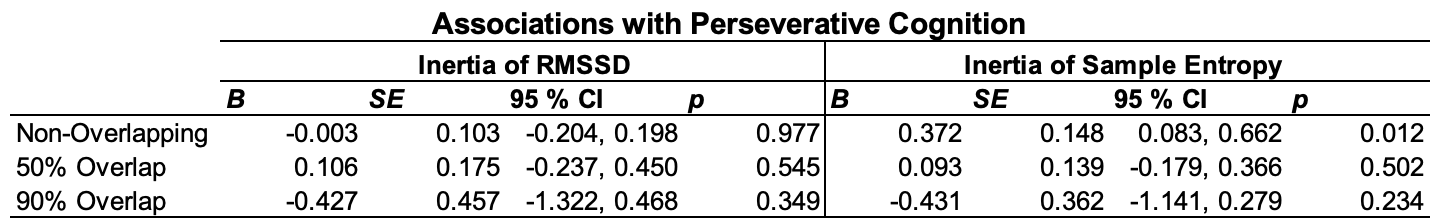
C)**

Note. RMSSD = root mean square of successive differences between R-R intervals.
